# Deficient Phagocytosis in Circulating Monocytes from Patients with COVID-19-Associated Mucormycosis

**DOI:** 10.1128/mbio.00590-23

**Published:** 2023-04-13

**Authors:** Bishnu Prasad Sinha, Priyanka Mehta, Md Asmaul Hoque, Purbita Bandopadhyay, Ayandip Nandi, Ipsita Saha, Anita Nandi Mitra, Asish Mondal, Boudhayan Bhattacharjee, Georgios Chamilos, Rajesh Pandey, Kaushik Basu, Dipyaman Ganguly

**Affiliations:** a IICB-Translational Research Unit of Excellence, CSIR-Indian Institute of Chemical Biology, Kolkata, India; b INtegrative GENomics of HOst-PathogEn (INGEN-HOPE) Laboratory, CSIR-Institute of Genomics and Integrative Biology, Delhi, India; c Academy of Scientific and Innovative Research, Ghaziabad, India; d Department of Pathology, Medical College, Kolkata, India; e Department of Microbiology, Medical College, Kolkata, India; f Department of General Medicine, Medical College, Kolkata, India; g Department of Tropical Medicine, School of Tropical Medicine, Kolkata, India; h Institute of Molecular Biology & Biotechnology, Foundation for Research & Technology - Hellas (IMBB FoRTH), Heraklion, Greece; i Department of Clinical Microbiology and Microbial Pathogenesis, School of Medicine, University of Crete, Heraklion, Greece; University of Florida College of Dentistry

**Keywords:** COVID-19, mucormycosis, monocytes, phagocytosis, cytokines, transcriptome

## Abstract

Cases of rhino-orbital mucormycosis in patients suffering from severe coronavirus disease 2019 (COVID-19) were reported in different parts of the world, especially in India. However, specific immune mechanisms that are linked to susceptibility to COVID-19-associated mucormycosis (CAM) remain largely unexplored. We aimed to explore whether the differential regulation of circulating cytokines in CAM patients had any potential pathogenic links with myeloid phagocyte function and susceptibility to mucormycosis. A small cohort of Indian patients suffering from CAM (N = 9) as well as COVID-19 patients with no evidence of mucormycosis (N = 5) were recruited in the study. Venous blood was collected from the patients as well as from healthy volunteers (N = 8). Peripheral blood mononuclear cells and plasma were isolated. Plasma samples were used to measure a panel of 48 cytokines. CD14^+^ monocytes were isolated and used for a flow cytometric phagocytosis assay as well as a global transcriptome analysis via RNA-sequencing. A multiplex cytokine analysis of the plasma samples revealed reduction in a subset of cytokines in CAM patients, which is known to potentiate the activation, migration, or phagocytic activity of myeloid cells, compared to the COVID-19 patients who did not contract mucormycosis. Compared to monocytes from healthy individuals, peripheral blood CD14^+^ monocytes from CAM patients were significantly deficient in phagocytic function. The monocyte transcriptome also revealed that pathways related to endocytic pathways, phagosome maturation, and the cytoskeletal regulation of phagocytosis were significantly downregulated in CAM patients. Thus, the study reports a significant deficiency in the phagocytic activity of monocytes, which is a critical effector mechanism for the antifungal host defense, in patients with CAM. This result is in concordance with results regarding the specific cytokine signature and monocyte transcriptome.

## OBSERVATION

A surge in cases of rhino-orbital mucormycosis, mainly caused by Rhizopus species, in patients suffering from severe coronavirus disease 2019 (COVID-19) was reported in India as well as in other parts of the world (1–3). Diabetes mellitus, corticosteroid pharmacotherapy, environmental exposure, and immune dysregulation are proposed as major predisposing factors for the development of COVID-19 associated mucormycosis (CAM) (1–3). Specific immune mechanisms linked to the susceptibility to CAM remain largely unexplored (3, 4). The inhibition of the growth of fungal conidia, following their uptake by tissue-resident and circulating phagocytes, is a crucial antifungal host defense mechanism against mucorales (5). Accordingly, defects in the numbers or functions of phagocytes markedly increase the susceptibility to mucormycosis (3, 5).

Severe COVID-19 disease is associated with a systemic hyperinflammation that is majorly characterized by an expansion of a dysfunctional myeloid cell subset and a cytokine storm involving the differential regulation of cytokines (6, 7). We wanted to explore whether cytokine dysregulations in COVID-19 have any potential pathogenic links with defects in myeloid phagocyte function and the related susceptibility to mucormycosis. Upon identifying the dysregulation of specific cytokines that are known to be mechanistically linked to myeloid cell activation and phagocytic functions, we explored the phagocytic dysfunction, if any, in the circulating monocytes.

**FIG 1:**
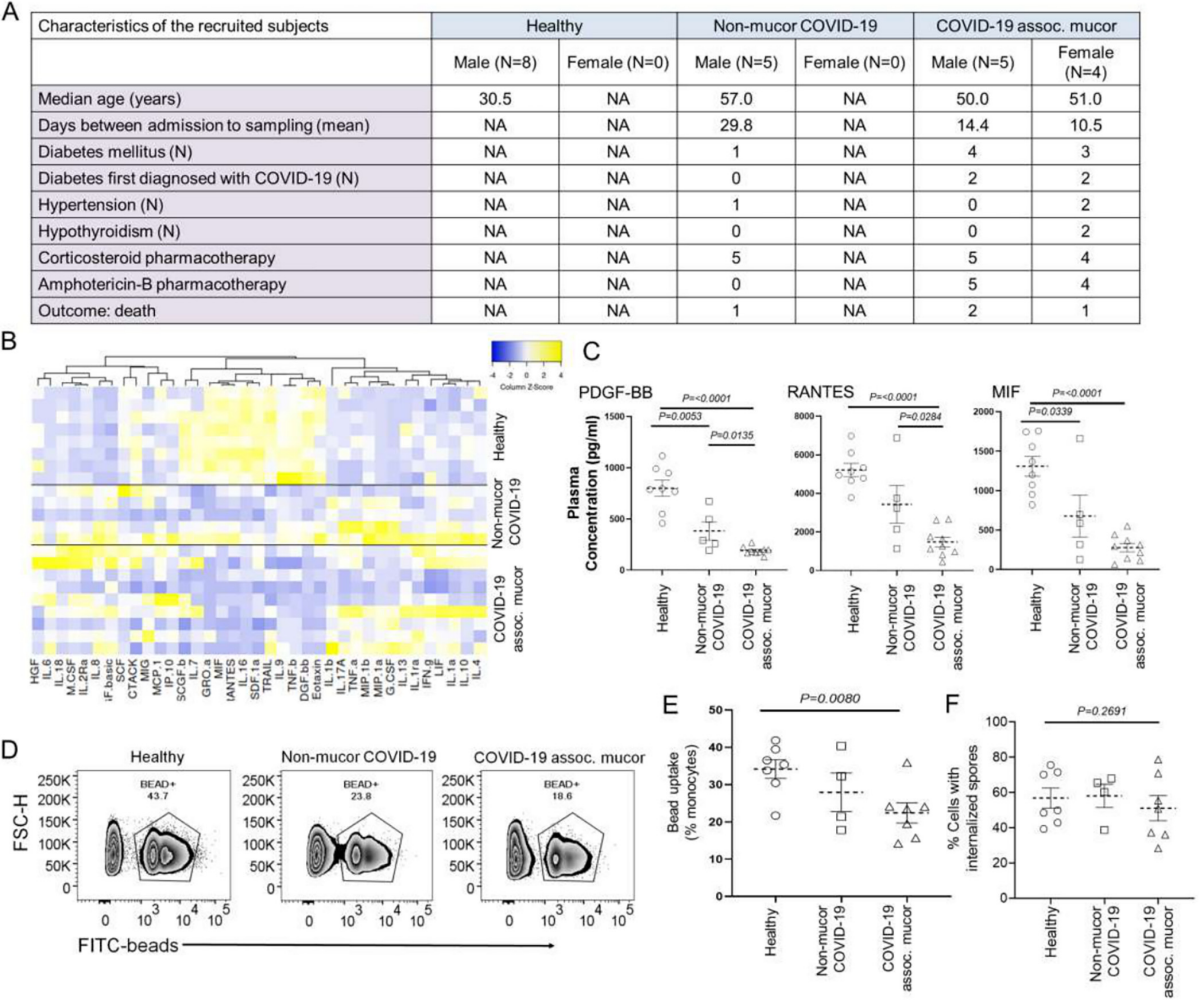
Deficient phagocytosis in circulating monocytes in COVID-19 patients with concomitant mucormycosis. (A) Key demographic features, major comorbidities, and relevant pharmacotherapies in the recruited subjects. (B) Heat map showing the clustering of the plasma abundance of 37 cytokines in all subgroups of recruited subjects. Complete linkage clustering was used, utilizing the Pearson distance measurement. (C) Relative plasma abundance of cytokines PDGF-BB, RANTES, and MIF, compared between healthy volunteers, COVID-19 patients without mucormycosis, and COVID-19 patients with concomitant mucormycosis. A two-tailed unpaired *t* test as well as a one-way analysis of variance (ANOVA) (*P* < 0.05) was performed. (D) Representative contour plots for the flow cytometric phagocytosis assay from three subgroups. (E) Percent monocytes showing the phagocytosis of the fluorescent latex beads, compared between healthy volunteers, COVID-19 patients without mucormycosis, and COVID-19 patients with concomitant mucormycosis. A two-tailed unpaired *t* test as well as a one-way analysis of variance (ANOVA) (*P* < 0.05) was performed. (F) Percent monocytes showing the phagocytosis of the fluorescent Rhizopus spores, compared between healthy volunteers, COVID-19 patients without mucormycosis, and COVID-19 patients with concomitant mucormycosis. A two-tailed unpaired *t* test as well as a one-way analysis of variance (ANOVA) (*P* < 0.05) was performed.

Patients suffering from CAM (N = 9) as well as COVID-19 patients with no evidence of mucormycosis (N = 5) who were admitted at the Department of Medicine, Medical College, Kolkata, India, were recruited into the study, which was approved by the Human Ethics Committee of Medical College, Kolkata (No. MC/KOL/IEC/NON-SPON/1102/06/2021). Venous blood was collected from the patients as well as from healthy volunteers (N = 8) upon obtaining written informed consent. Peripheral blood mononuclear cells (PBMCs) and plasma were cryostored. Key details for the recruited individuals are provided in Fig. 1A. Seven out of nine CAM patients were diabetic, and four of them had their first diagnosis of diabetes after their admission for COVID-19. One patient out of the five COVID-19 patients without mucormycosis also had diabetes. Three out of nine CAM patients met fatal outcomes.

### Comparison of differential cytokine abundance in plasma.

Plasma was used to measure a panel of cytokines for all subjects (Bio-Plex Pro 48-Plex Assay, Bio-Rad), and 37 cytokines were present in detectable levels. The characteristic dysregulation of systemic cytokine abundance in severe COVID-19 patients, which was previously widely reported (6, 7), was also predominant in the CAM patients, as expected (Fig. 1B). Interestingly, a subset of cytokines (i.e., the platelet-derived growth factor-BB [PDGF-BB], the chemokine CCL5 [also known as regulated upon activation, normal T cell expressed, and secreted or RANTES], and the macrophage migration inhibitory factor [MIF]), showed a significant reduction in abundance in the CAM patients, compared to the control COVID-19 patients (Fig. 1C). These three cytokines are known to potentiate the activation, migration, or phagocytic activity of myeloid lineage cells (8–11).

### Comparison of phagocytic functions of circulating monocytes.

Efficient phagocytosis by myeloid lineage cells, especially circulating monocytes and neutrophils, is a key mechanism for antifungal innate immunity (3). The significant reduction of PDGF-BB, RANTES, and MIF, in the CAM patients led us to explore the phagocytic activity in the circulating monocytes from the recruited COVID-19 patients, with or without mucormycosis, compared to those from healthy volunteers. This was done via flow cytometry-based phagocytic assay using fluorescent latex beads (see Supplemental Material). The flow cytometric assay for phagocytosis was done using CD14^+^ monocytes, isolated from PBMCs, for 7 samples from healthy participants, 4 samples from patients with COVID-19 without mucormycosis, and 7 samples from CAM patients, using 1 μm carboxylate-modified polystyrene yellow green latex beads (Sigma). We found that, compared to healthy individuals, CD14^+^ monocytes isolated from CAM patients were significantly deficient in phagocytosing latex beads (Fig. 1D and E). Though not significant in the small cohort of patients that we studied, this deficiency in monocyte phagocytosis was more marked than that observed in the COVID-19 patients without mucormycosis (Fig. 1E). To further explore the phagocytic activity of monocytes in response to a more physiological stimulus, we did a similar flow cytometry-based phagocytosis assay (see Supplemental Material), utilizing UV-inactivated spores that were isolated from agarose cultures of a wild type Rhizopus delemer strain (12, 13). Also, in this experiment, the defective phagocytic activity in the circulating monocytes from the CAM patients was apparent; however, it was not significant in this small cohort (Fig. 1F). The physiological content of the pattern recognition receptor ligands in the fungal spores might also have played a role in the higher efficiency of the CAM monocytes in phagocytosing them, in comparison to the latex beads.

### Comparison of the gene expression landscape of the circulating monocytes.

**FIG 2:**
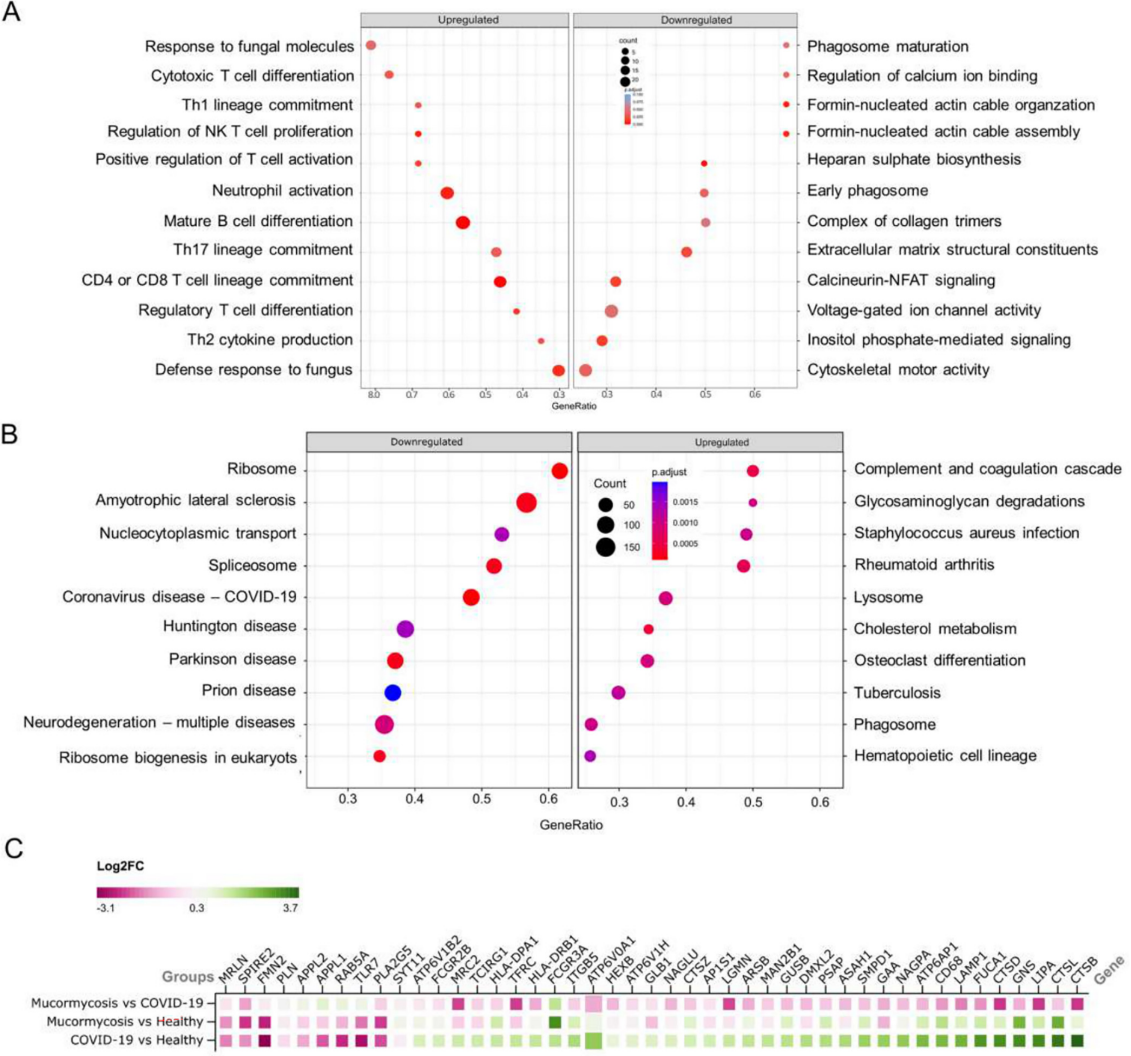
The monocyte transcriptomes reveal the downregulation of phagocytosis-related pathways. (A) The dot plot represents the biological processes that are upregulated and downregulated in the COVID-19 with mucormycosis group. The size of the dot represents the number of genes enriched in the processes, and the color of the dot represents the significance (adjusted *P* value) of the processes. (B) The dot plot represents biological processes that are upregulated and downregulated in the COVID-19 group, compared to those of healthy monocytes. The size of the dot represents the number of genes enriched in the process, and the color of the dot represents the significance (adjusted *P* value) of the process. (C) The plot represents a selection of differentially expressed genes that are relevant to phagocytic functions in monocytes, compared between different groups of subjects, as indicated.

To further characterize this monocyte dysfunction, we performed next-generation sequencing on the cellular RNA from purified monocytes from COVID-19 patients without mucormycosis (N = 5), CAM patients (N = 8), and healthy individuals (N = 3), see Text S1. Analyses of differentially regulated genes revealed that pathways related to systemic inflammation and the antifungal immune response (e.g., response to fungal molecules, Th17 lineage commitment, neutrophil activation, etc.) were upregulated in the monocytes from the CAM patients, compared to healthy monocytes, as expected (Fig. 2A–C; Tables S1 and S2). On the other hand, a number of pathways related to endocytosis, phagosome maturation, and cytoskeletal regulation linked to phagocytosis were significantly downregulated in the transcriptomes of the CAM monocytes, compared to those of the healthy monocytes. On the other hand, the monocytes from the COVID-19 patients, compared to the healthy monocytes, did not show such prominent downregulation of the pathways related to phagocytic functions (Fig. 2B). Analyzing the fold changes among the groups, a selection of relevant genes also demonstrated a CAM-specific downregulation of the genes that are crucially involved in phagocytic activity (Fig. 2C). The genes downregulated in the CAM monocytes, compared to the monocytes from the COVID-19 patients, include those for the mannose receptor (MRC2), lysosomal cathepsins (CTSB, CTSD, CTSL, CTSZ), legumain (LGMN), and lysosomal acid lipase A (LIPA), which were observed to be rather upregulated in the COVID-19 patients, compared to the healthy monocytes (Fig. 2C). This disruption in the expression of the C-type lectin receptors and the key lysosomal enzymes also plausibly points to a functional deficiency of phagolysosomal proteolysis and lipolysis in the monocytes from the CAM patients.

### Discussion.

In the present study, we investigated a small cohort of COVID-19 patients who had concomitant afflictions with mucormycosis. Finding a markedly reduced plasma abundance of cytokines, such as PDGF-BB (which is known to potentiate phagocytosis in myeloid cells), RANTES (which is known to activate migration, phagocytotic, and intraphagosomal lytic mechanisms in myeloid cells), and MIF (which is a well-known activator of monocytes and macrophage functions), led us to examine the phagocytic activity in the circulating monocytes from the CAM patients (8–11). The phagocytic activity and cellular transcriptomes of the circulating monocytes revealed that a deficiency in the phagocytic activity of circulating monocytes may underlie the susceptibility for contracting the fungal infection.

Phagocytosis is a key innate immune mechanism in anti-fungal immunity, and monocytes and macrophages play a major role (3, 4). The evasion of phagocytosis through surface sialoglycoproteins is reported to be a potential immune escape mechanism for fungal pathogens (14). The monocyte transcriptomes analyzed in our study were also revealing, as a marked downregulation of pathways related to phagocytosis, phagosome maturation, and cytoskeletal regulation was registered, despite the upregulation of other pathways that are known to positively affect antifungal innate immunity.

MIF, which is a cytokine that showed a markedly reduced plasma abundance in the CAM patients, is also known to interfere with myeloid cell dysfunction in the context of corticosteroids (15). Corticosteroid therapy has been proposed to underlie the susceptibility to CAM. Thus, these cytokines may have predictive potential for such a susceptibility during corticosteroid therapy, which warrants further exploration in a larger cohort.

Of note here, in a recent study, transcriptomic analyses of bronchoalveolar lavage samples from COVID-19-associated aspergillosis patients also revealed dysregulations in neutrophil abundance and in the transcriptional program involved in phagocytic functions (16). Another study reported T cell dysfunction in response to Rhizopus and Aspergillus antigens in COVID-19 patients, pointing to another plausible immune evasion mechanism leading to a susceptibility to a fungal infection in COVID-19, independent of the influence of corticosteroid pharmacotherapy (17).

Major limitations of our study stem from the small cohort size, which precluded the exclusion of all potential confounding factors (e.g., ongoing pharmacotherapy, comorbid clinical conditions, effect of biological sex, etc.) of the susceptibility to mucormycosis infection. In addition, there was an issue of not having balanced sex representation in the groups, especially among the healthy group and the group of COVID-19 patients without mucormycosis. This limitation came from the emergent nature of the study, which was done on samples collected from COVID-19 patients that were admitted at the same clinical institution during the same time period. This decision was made to minimize the confounders related to contemporaneously adopted standard of care and environmental influences. Despite these unavoidable limitations, the present study, which points to dysfunctional monocyte phagocytosis, provided crucial insight into yet another plausible immune evasion mechanism that is operative in COVID-19 and increases the susceptibility to mucormycosis and, perhaps, to other fungal infections.

10.1128/mbio.00590-23.1 TEXT S1 Supplemental methods. Download Text S1, PDF file, 0.5 MB. Copyright © 2023 Sinha et al. 2023 Sinha et al. https://creativecommons.org/licenses/by/4.0/ This content is distributed under the terms of the Creative Commons Attribution 4.0 International license.

10.1128/mbio.00590-23.2 TABLE S1 Differential enrichment of gene expression pathways among the different study groups. Download Table S1, XLSX file, 0.7 MB. Copyright © 2023 Sinha et al. 2023 Sinha et al. https://creativecommons.org/licenses/by/4.0/ This content is distributed under the terms of the Creative Commons Attribution 4.0 International license.

10.1128/mbio.00590-23.3 TABLE S2 Differentially expressed genes in circulating monocytes among the study groups. Download Table S2:, XLSX file, 4.9 MB. Copyright © 2023 Sinha et al. 2023 Sinha et al. https://creativecommons.org/licenses/by/4.0/ This content is distributed under the terms of the Creative Commons Attribution 4.0 International license.

### Data availability.

The data is available under the BioProject Id: PRJNA875643 and Accession IDs (SRR24068857 to SRR24068853 and SRR21386287 to SRR21386277) at NCBI (https://www.ncbi.nlm.nih.gov/).
