# Supplementary material for: Deficient Phagocytosis in Circulating Monocytes from Patients with COVID-19-Associated Mucormycosis
Source: mBio. 2023 Apr 13;14(3):e00590-23. doi: 10.1128/mbio.00590-23 (PMC10294693; doi:10.1128/mbio.00590-23)
Supplement: TEXT S1 [file mbio.00590-23-s0001.pdf]

SUPPLEMENTAL METHODS

Subject recruitment

Patients suffering from CAM (N=9) as well as COVID-19 patients with no evidence of mucormycosis (N=5), admitted at the Department of Medicine, Medical College, Kolkata, India, were recruited in the study, approved by Human Ethics Committee of Medical College, Kolkata (No. MC/KOL/IEC/NON-SPON/1102/06/2021). All COVID-19 patients received corticosteroids (dexamethasone 0.1- 0.4 mg/ kg x 5-7 days or equivalent dose of other steroids) and all CAM patients were treated with intravenous amphotericin-B (5mg/kg for 21 days).

Plasma cytokine analysis

Plasma abundance was measured for 48 cytokines using Bio-Plex Pro 48-Plex Assay, Bio-Rad. The analytes, measured in plasma diluted 1:3 in sample diluent, are: cutaneous T cell-attracting chemokine (CTACK), fibroblast growth factor (FGF) basic, eotaxin, granulocyte-macrophage colony-stimulating factor (GM-CSF), granulocyte colony-stimulating factor (G-CSF), M-CSF, interferon-γ (IFN-γ), IFN-α2, IFN-γ–inducible protein-10 (IP-10), interleukin-1β (IL-1β), IL-1ra, IL-1α, IL-2, IL-2Rα, IL-3, IL-4, IL-5, IL-6, IL-7, IL-8, IL-9, IL-10, IL-12 (p70), IL-12 (p40), IL-13, IL-15, IL-16, IL-17A, IL-18, growth-related oncogene-α (GRO-α), hepatocyte growth factor (HGF), leukemia inhibitory factor (LIF), monocyte chemoattractant protein-3 (MCP-3), macrophage-inflammatory protein-1α (MIP-1α), MIP-1β, MCP-1, monokine induced by interferon-γ (MIG), migration inhibitory factor (MIF), nerve growth factor-β (NGF-β), stem cell growth factor-β (SCGF-β), stem cell factor (SCF), stromal cell-derived factor-1α (SDF-1α), platelet-derived growth factor-BB (PDGF-BB), regulated upon activation normal T-cell expressed and secreted (RANTES), tumor necrosis factor-α (TNF-α), TNF-β, TNF-related apoptosis-inducing ligand (TRAIL) and vascular endothelial growth factor (VEGF). The absolute concentrations of the cytokines were measured against manufacturer-supplied standards in the Bio-Plex software.

Flow cytometric phagocytosis assay

$CD14^+$ monocytes were purified from PBMCs using anti-CD14 magnetic microbeads (Miltenyi Biotec). Fraction of the purified monocytes was used to check for phagocytic function, while another fraction was cryostored for RNA isolation. Flow cytometric assay for phagocytosis was done using 1µm carboxylate-modified polystyrene yellow green latex beads (Sigma). After incubation with beads (2.5%

stock aqueous suspension diluted 1:50 in PBS) with 10^5 monocytes at 37°C with 5% CO2 for 3 hrs cells were washed before flow cytometry. For assessing phagocytosis of fungal spores UV-inactivated conidia from a wild type strain *Rhizopus delemar* 99-880, from a brain isolate obtained from the University of Texas Health Science Center at San Antonio, were used (references in main text: 17, 18). The strain was grown on Yeast extract agar glucose agar plates for 3 days at 37 °C. Fungal conidia (spores) were harvested by gentle shaking in the presence of sterile 0.1% Tween-20 in phosphate-buffered saline (PBS), washed twice with PBS, filtered through a 40 µm pore size cell strainer (Falcon) to separate conidia from contaminating mycelium, counted by a hemocytometer, and suspended at a concentration of 10^7 and 10^8 spores/ml. Inactivation of *Rhizopus* conidia was done by exposure to UV light (1 h at room temperature). For fluorescence labeling, 10^6 conidia were stained in 100 µl PBS containing 100 µg/ml Fluorescent Brightener 28 (Sigma-Aldrich, cat no. 475300) and 0.1 M NaHCO3 for 30 min protected from light in a bench-top rotator. Then the labeled conidia were washed three times with PBS and the concentration was adjusted to 10^7 or 10^8 conidia/ml. Flow cytometric assay for phagocytosis was done by incubation with spores (stock suspension diluted 1:50 in PBS) with 10^5 monocytes at 37°C with 5% CO2 for 3 hrs cells were washed before flow cytometry.

Monocyte transcriptome

RNA sequencing on the total cellular RNA from purified CD14$^+$ monocytes was done on Nextseq 2000 using P2 flowcell at 2x151 read length and loading concentration of 650 pM. The raw sequencing reads were quality checked using Fastqc (https://www.bioinformatics.babraham.ac.uk/projects/fastqc/) which were filtered and trimmed with Trimmomatic (v.0.39) to remove low quality bases[19].

The filtered reads were then aligned to the reference Human genome (assembly GRCh38.104) using Salmon (v1.4.0)[20]. The quantification generated from Salmon were then imported to R environment using tximport package and differential gene identification was performed using DESeq2 (10.1186/s13059-014-0550-8) (v.1.30.1)[21]. The differential gene expression analysis was performed between Mucormycosis/Infected vs Healthy, COVID-19 vs Healthy and Mucormycosis vs COVID-19. Differentially expressed genes with p value <0.05 and log2 fold change of 1.5 were called as significant.

Functional enrichment of DEGs was performed using Enrichr against Gene Ontology (GO) database and statistical significance was calculated using Fisher's Exact test[22]. Pathways related to infection and with a p value < 0.01 were considered. The pathways were plotted using the ggplot2 (Wickham, H., 2006. An introduction to ggplot: An implementation of the grammar of graphics in R. Statistics, pp.1-8.) R package and rawgraphs (https://app.rawgraphs.io/).

Statistics

All statistical analyses for functional studies were done in GraphPad Prism software and the tests done are indicated in the figure legends. Statistical analyses for the RNA-seq data were done as described above.

Additional references:

19. Bolger AM, Lohse M, Usadel B. Trimmomatic: a flexible trimmer for Illumina sequence data. Bioinformatics. 2014 Aug 1;30(15):2114-20. doi: 10.1093/bioinformatics/btu170.

20. Patro R, Duggal G, Love MI, Irizarry RA, Kingsford C. Salmon provides fast and bias-aware quantification of transcript expression. Nat Methods. 2017 Apr;14(4):417-419. doi: 10.1038/nmeth.4197.

21. Soneson C, Love MI, Robinson MD. Differential analyses for RNA-seq: transcript-level estimates improve gene-level inferences. F1000Res. 2015 Dec 30;4:1521. doi: 10.12688/f1000research.7563.2.

22. Chen EY, Tan CM, Kou Y, Duan Q, Wang Z, Meirelles GV, Clark NR, Ma'ayan A. Enrichr: interactive and collaborative HTML5 gene list enrichment analysis tool. BMC Bioinformatics. 2013 Apr 15;14:128. doi: 10.1186/1471-2105-14-128.
